# Operando Spectroscopic Studies of Cu–SSZ-13 for NH_3_–SCR deNOx Investigates the Role of NH_3_ in Observed Cu(II) Reduction at High NO Conversions

**DOI:** 10.1007/s11244-018-0888-3

**Published:** 2018-01-19

**Authors:** Alex G. Greenaway, Ines Lezcano-Gonzalez, Miren Agote-Aran, Emma K. Gibson, Yaroslav Odarchenko, Andrew M. Beale

**Affiliations:** 1 Department of Chemistry, UCL, 20 Gordon Street, London, WC1H 0AJ UK; 2 Research Complex at Harwell, Rutherford Appleton Laboratory, Harwell, Didcot, OX11 0FA UK

**Keywords:** Catalysis, Operando, XANES, Cu–SSZ-13, Zeolite

## Abstract

The small pore zeolite chabazite (SSZ-13) in the copper exchanged form is a very efficient material for the selective catalytic reduction by ammonia (NH_3_) of nitrogen oxides (NOx) from the exhaust of lean burn engines, typically diesel powered vehicles. The full mechanism occurring during the NH_3_–SCR process is currently debated with outstanding questions including the nature and role of the catalytically active sites. Time-resolved operando spectroscopic techniques have been used to provide new level of insights in to the mechanism of NH_3_–SCR, to show that the origin of stable Cu(I) species under SCR conditions is potentially caused by an interaction between NH_3_ and the Cu cations located in eight ring sites of the bulk of the zeolite and is independent of the NH_3_–SCR of NOx occurring at Cu six ring sites within the zeolite.

**Electronic supplementary material:**

The online version of this article (10.1007/s11244-018-0888-3) contains supplementary material, which is available to authorized users.

## Introduction

**Fig. 1:**
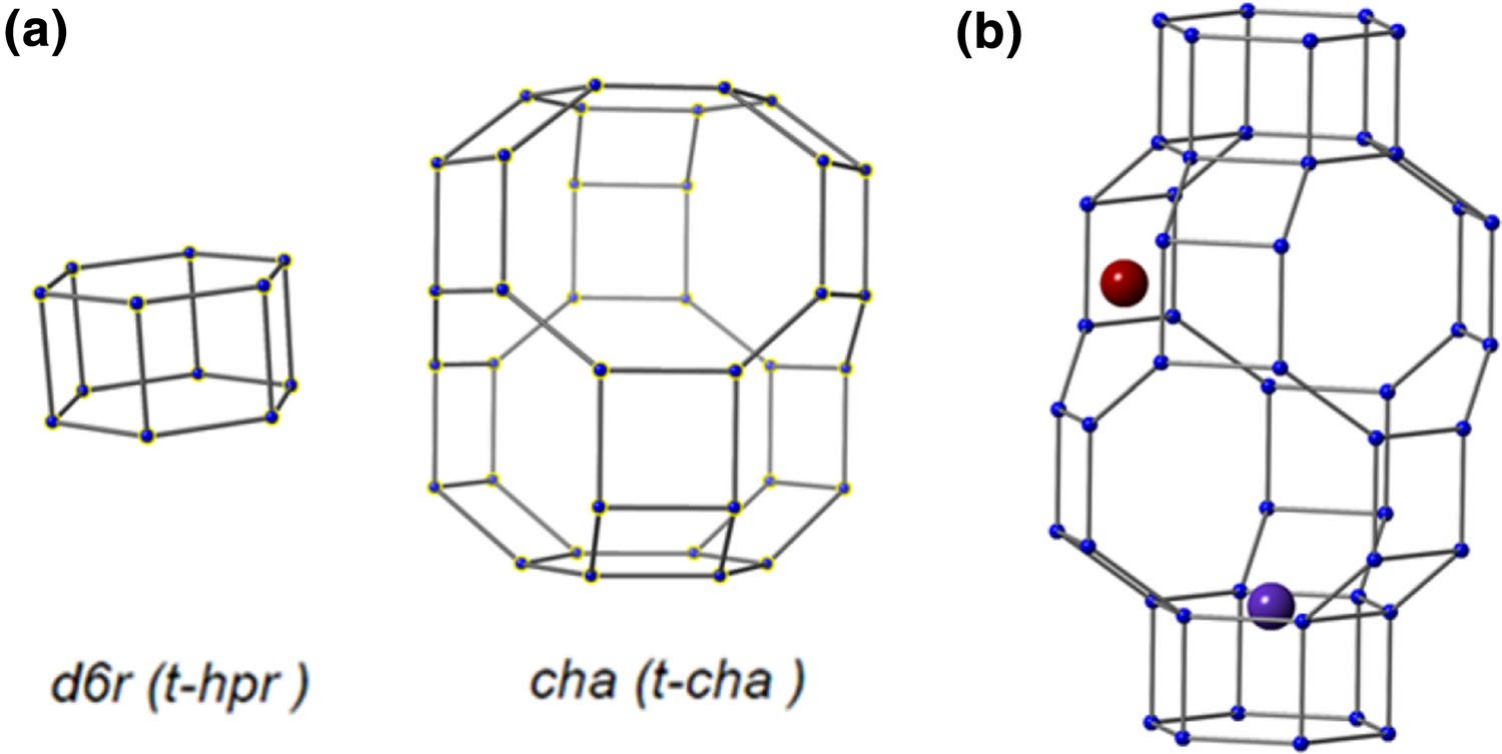
**a** Cage representation of the two cage types, d6r and cha, present in chabazite, **b** schematic representation of two different Cu cations sites that can be present in the dehydrated form of SSZ-13, purple atom in face of d6r, red position in face of 8r in cha cage

Selective catalytic reduction using ammonia (NH_3_–SCR) is regarded as an efficient way to prevent the emission of nitrogen oxides (NO, NO_2_) from the exhaust gas of lean burn engines, especially those of diesel powered vehicles [1–3]. Initially vanadia/titania systems [4] were considered the most -promising SCR catalysts, however, due to issues with hydrothermal durability and concerns over vanadia waste (particularly in the US), metal-exchanged zeolites became more widely studied and used in vehicle applications. Originally Cu/Fe ZSM-5 [5, 6] and Cu/Fe-beta [7, 8] were studied for NH_3_–SCR for deNOx, however, issues relating to thermal stability and degradation (caused by temperature spikes in the catalyst bed due to the combustion of unburned hydrocarbons) [9] motivated research in to the exploration of new, more stable systems including small-pore metal-exchanged zeolites, such as SSZ-13 (CHA) [10, 11], zeolite A (LTA) [12] and SSZ-39 (AEI) [13] for SCR applications. Cu–SSZ-13 is the subject of a considerable amount of research as it has both a higher activity, selectivity and is less susceptible to thermal degradation than ZSM-5 and beta based systems [14–16], chabazite is a small pore zeolite (maximum ring size 8 t-sites) composed of double 6 ring (d6r) and chabazite (cha) cages with the d6r cages in an AABBCC packing sequence (see Fig. 1).

In the hydrated form, there are a number of positions that can be adopted by Cu(II) cations within the porous material, upon dehydration the Cu(II) cations migrate to sites in which the cations can maximise their interactions with the framework oxygens [16]. There are two highly preferable sites within the framework, in 8r window connecting cha cages and in the corners of the d6r cages, [17–19]. At low temperatures, the 8r site is preferable, enabling the formation of large Cu^2+^ species such as [Cu(NH_3_)]^2+^ when in the presence of NH_3_ [20], however, upon a modest temperature increase (250 °C) the d6r window becomes favourable [21]. While there have been many studies in to Cu–SSZ-13 there are still several debated questions regarding the catalytic mechanism which namely relate to the nature and position of the active sites within the framework.

Operando experiments, in which the spectroscopic/diffraction techniques are being applied under conditions relevant to the catalytic process being studied is coupled to measurement of catalytic activity and selectivity through analysing the products of the reaction, has proven to be a highly useful tool to provide insight into catalysts under reaction conditions. Cu–SSZ-13 has been studied via many techniques under operando conditions and the results have been outlined in a recent review [9]. Operando experiments for NH_3_–SCR are however inherently complex to set up, perform and analyse, due to several factors and or limitations which may be out of the control of the scientists performing the experiments. These can include apparatus limitations, available flow from mass flow controllers (MFCs), wall thickness of reaction cell used, number of gas streams available and gas concentrations permissible under HSE at the facility where the experiment is being performed. These factors can all influence the composition of the gas mixture at which the operando experiment is performed. In a real-world environment, a typical lean burn diesel vehicle catalyst would be operating under conditions that are approximately [22]; A gas mixture made up of N_2_ mixture containing 350 ppm NO, 350 ppm NH_3_, 14% O_2_, 5% CO_2_, and 5% H_2_O at a gas hourly space velocity (GHSV) of 30,000 h^−1^. Furthermore both the composition, temperature and rate of gas stream can all vary during operation such as under significant acceleration events. As deviations in conditions may result in the changes of the chemistry that is occurring within the materials, care needs to be taken when selecting conditions under which to perform experiments as well as when interpreting results from operando experiments and linking the results to the catalytic process occurring.

The results reported in this paper focus on operando and in-situ XANES experiments of two Cu–SSZ-13 materials (Cu–SSZ-13-a, 75% Cu(II) ion exchange; Cu–SSZ-13-b, 100% Cu(II) ion exchange) which have been previously shown to be highly active for NH_3_–SCR [21, 23]. Cu–SSZ-13-a contains Cu ion exchanged in to 75% of the available H^+^ sites. Cu–SSZ-13-b has 100% Cu ion exchanged in to available H^+^ sites. Upon activation in dry air at 250 °C Cu–SSZ-13-a shows occupancy of Cu ions in both the 6r and 8r sites (see Fig. 1) whereas Cu–SSZ-13-b exhibits almost zero occupancy in the 8r site as all Cu ions have migrated to 6r sites. The experiments reported observe some Cu(I) present in the samples at experimental conditions relevant to high NO conversions. Through performing various experiments under different gas conditions, at different GHSV and with different catalysts, it is shown that this observation is due to an NH_3_ interaction which occurs at the more highly reducible Cu cation positioned in the 8r site of the zeolite.

## Experimental

### Preparation and Characterisation of Catalysts

SSZ-13 zeolite (Si/Al = 13) was synthesized as described previously, using *N,N,N*-trimethyladamantammonium hydroxide as a structural directing agent in a fluoride media, under static hydrothermal conditions [13, 21, 23]. The proton form of the zeolite is obtained by calcining the sample in air by heating at 1 °C min^−1^ to 120 °C, held for 2.5 h and then at 4 °C min^−1^ to 550 °C, held for 10 h. The copper exchanged form was prepared using a wet ion exchange method in which typically an amount of the H-SSZ-13 would be added to an aqueous solution of copper sulphate (50 ml of a 0.1 M solution of CuSO_4_ per gram of zeolite) and heated at 80 °C for 2 h whilst stirring. The product is then recovered by vacuum filtration, washed with copious amounts of water, dried overnight at 80 °C and calcined in air using the procedure outlined above (see ESI for further details). Cu–SSZ-13-a (2.92 wt% Cu; Si/Al = 13) is a sample which was prepared by following this procedure once and the material is 75% Cu ion exchanged in to available H^+^ sites. Cu–SSZ-13-b (3.86 wt% Cu: Si/Al = 12) was prepared by sequentially performing 4 ion exchange and calcinations on a sample obtained from Johnson Matthey UK, and is a material in which 100% Cu ion exchanged in to available H^+^ sites has occurred. The materials consist of well-defined rhombohedral crystals with good phase purity and crystallinity and contain isolated Cu(II) species upon dehydration, with no evidence of particles of copper or copper oxide present in the UV–vis spectra (For further information about synthesis, characterisation and catalytic testing see ESI). Prior to performing operando spectroscopic analysis, the zeolites were pressed in to pellets (8 mm bore, 1.5 tonne pressure), broken up and sieved, retaining the 250–440 µm fraction for experiments.

### Operando XAS

Cu K-edge XAS studies were performed at the Diamond Light Source (DLS), UK, [24] on beamline B18 and at the Swiss Light Source (SLS) at the Paul Scherrer Institute, Switzerland, on the SuperXAS beamline [25]. At the DLS, measurements were performed in transmission mode using ion chamber detectors with a fast scanning (QEXAFS) Si(111) double crystal monochromator. Each spectrum took 60 s to acquire with a Cu foil placed between It and Iref. At the SLS the Si(111) crystal of the quickXAS monochromator was oscillated at 5 Hz and the spectra averaged over 1 min. At both beamlines, the sieved zeolite was placed in to a quartz capillary based gas flow cell (3 mm diameter at diamond, 0.8 mm diameter at SLS), the zeolite material was placed in the reactor cell between two quartz wool plugs to prevent physical movement of the material under flowing gas conditions. The capillary was sealed in to the gas flow cell by screw fittings to ensure a gas tight connection and a thermocouple was inserted into the end of the cell such that it reached the centre point of the catalyst bed. The cell was mounted into the beamline so that the beam was focussed towards the front section of the catalyst bed, while ensuring the beam was fully focussed within the sample (beam size 0.5 × 0.5 mm at SLS, 0.05 × 0.05 mm at DLS), temperature control was achieved using hot air blowers and attached to heated gas lines controlled by MFCs, the outlet of the gas cell was connected to a mass spectrometer (MKS mass spectrometer at DLS, Hiden at the SLS) that was used to follow signals of m/z 2 (H_2_), 4 (He), 18 (H_2_O), 28 (CO, N_2_), 30 (NO), 32 (O_2_), 44 (N_2_O/CO_2_), and 46 (NO_2_).

Prior to the operando experiments, the calcined catalysts were subjected to high temperature activation in an O_2_ atmosphere. Samples were heated to 400 °C at 10 °C min^−1^ and held at this temperature until no more changes were observed in the XAS spectra, the sample was then allowed to attain the desired temperature. At DLS a temperature ramp experiment was conducted during which the sample was heated from 150 to 600 °C at 5 °C min^−1^, during this temperature ramp the sample was exposed to an atmosphere of NH_3_ (3 ml min^−1^, 5% in He), NO (15 ml min^−1^, 1% in He), O_2_ (28.571 ml min^−1^, 17.5% in He) and He (3.428 ml min^−1^) giving a total flow of 50 ml min^−1^, 3000 ppm of NH_3_ and NO in 10% O_2_ over a packed bed, of 8 mm in length with a radius of 1.5 mm, giving a GHSV of 53,000 h^−1^. NO conversion was calculated as a function of intensity of NO mass spec signal and the intensity of equilibrated NO mass spec signal at low temperature $\left[ {\left( {\left( {{{\text{I}}_{{\text{ref}}}} - {{\text{I}}_{{\text{temp}}}}} \right){\text{/}}{{\text{I}}_{{\text{ref}}}}} \right)\times100\%} \right].$ At the SLS experiments based on varying the GHSV, at a constant temperature of 250 °C, were conducted under conditions for both NH_3_–SCR and NH_3_ + O_2_. During the NH_3_–SCR experiments the zeolite catalysts were exposed to the following gas conditions, NH_3_ (2.5 ml min^−1^, 1% in N_2_), NO (2.5 ml min^−1^, 1% in N_2_), O_2_ (2 ml min^−1^, 100%) and N_2_ (12.8 ml min^−1^) giving a total flow of 19.8 ml min^−1^, 1262 ppm of NH_3_ and NO in 10% O_2_ over a packed bed of 7 mg of zeolite (density of zeolite = 0.526 g cm^−3^_,_ volume of bed = 0.0133 cm^−3^), giving a GHSV of 90,000 h^−1^. Under a second experiment at higher GHSV the samples were exposed to NH_3_ (6.5 ml min^−1^, 1% in N_2_), NO (6.5 ml min^−1^, 1% in N_2_), O_2_ (6 ml min^−1^, 100%) and N_2_ (32 ml min^−1^) giving a total flow of 50 ml min^−1^, 1262 ppm of NH_3_ and NO in 10% O_2_ over a packed bed of 7 mg of zeolite, giving a GHSV of 225,000 h^−1^. The two GHSV experiments were repeated under conditions in which both NH_3_ and O_2_ were present, in which the gas composition remained the same with the removal of the NO and addition of extra N_2_ to ensure the same GHSV were maintained (full details of the gas flow calculations can be found in the ESI). EXAFS data processing was performed using IFEFFIT [26] with the Horae package [27] (Athena and Artemis).

## Results and Discussion

### Operando XAS NH_3_–SCR Temperature Ramp Experiment on Cu–SSZ-13-a

**Fig. 2:**
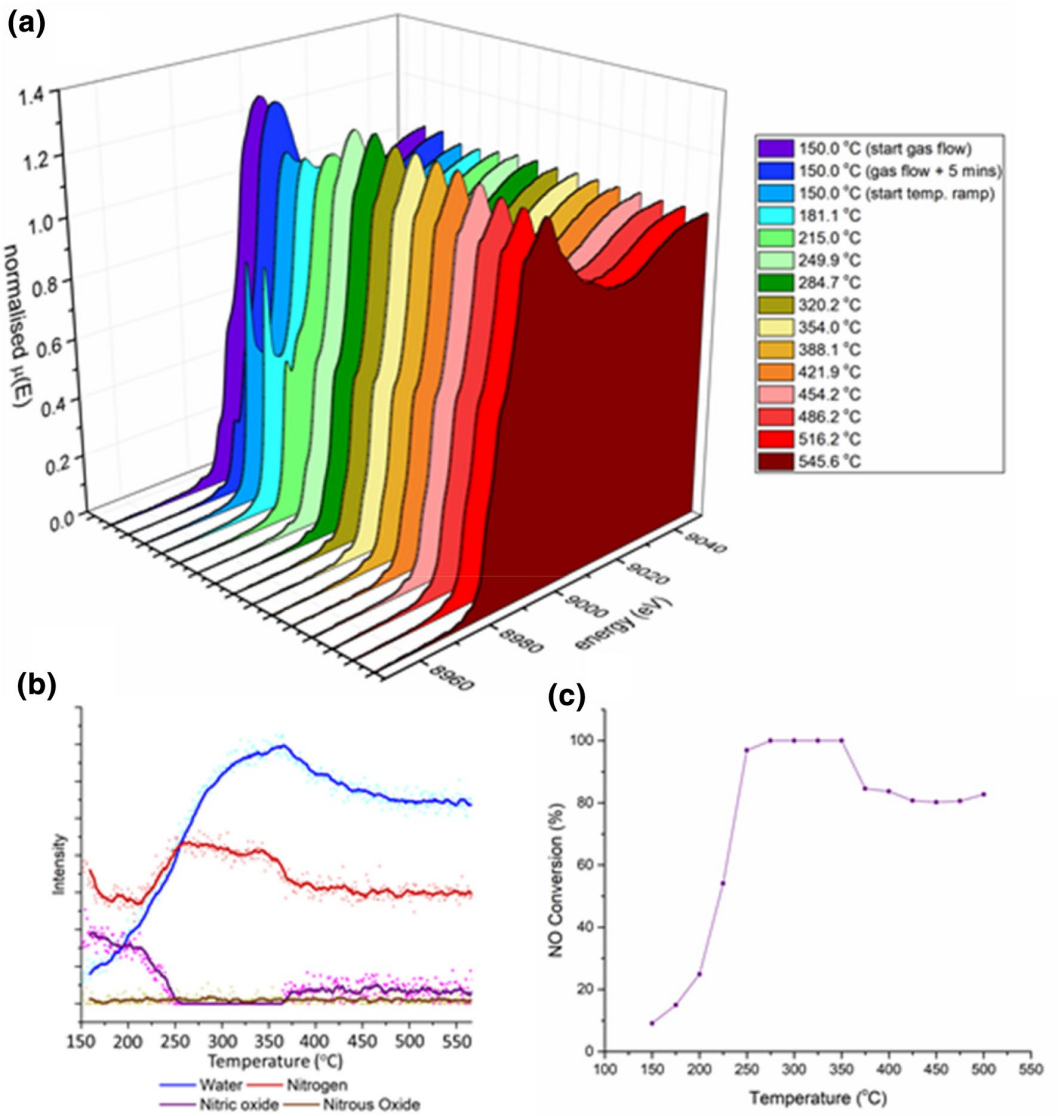
**a** XANES spectra collected under NH_3_–SCR of NO conditions (3000 ppm of NH_3_, 3000 ppm NO, 100,000 ppm O_2_, make up He, GHSV of 53,000 h^−1^) during a temperature ramp experiment (5 °C min^−1^), **b** mass spectroscopy data collected from the out gas of the reaction cell, time = 0 s represents initial exposure of experimental gas composition, **c** NO conversion as a function of temperature, data collected at DLS (B18)

**Fig. 3:**
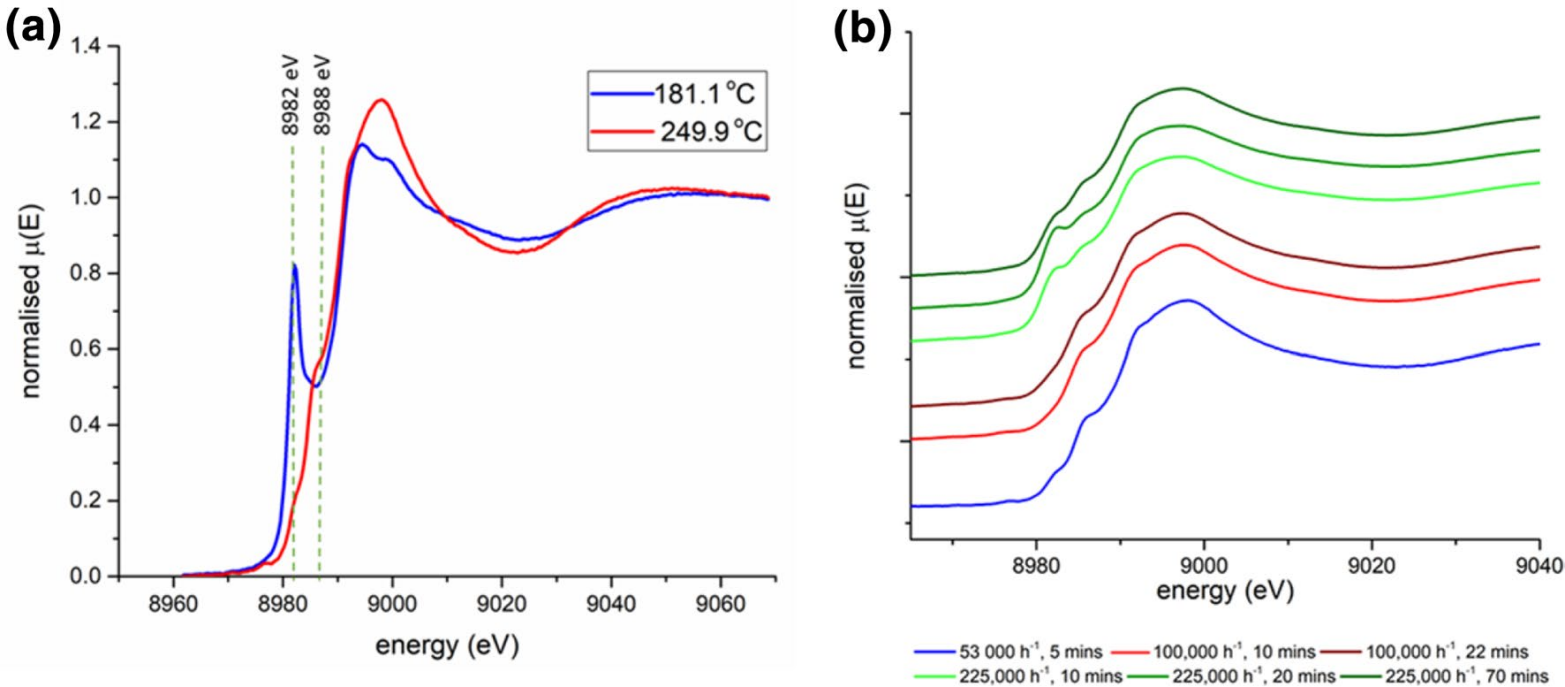
**a** XANES spectra recorded under NH_3_–SCR of NO conditions (3000 ppm of NH_3_, 3000 ppm NO, 100,000 ppm O_2_, make up He, GHSV of 53,000 h^−1^) for Cu–SSZ-13-a at 181 and 250 °C. Spectra at 181 °C show prominent Cu(I) species which is markedly decreased by 250 °C at 8982 eV. Spectra at 250 °C also shows a small pre-edge shoulder at 8988 eV assigned to the Cu^2+^ 1s/4p transition, (data recorded at DLS), ** b** XANES data recorded at different GHSV, blue spectra recorded at 53,000 h^−1^ (data recorded at DLS) red spectra represent an experiment conducted at approximately 100,000 h^−1^ and green spectra an experiment at 225,000 h^−1^ (data recorded at SLS, 1262 ppm of NH_3_, 1262 NO, 100,000 ppm O_2,_ make up N_2_)

A temperature ramp experiment was conducted on an activated sample of Cu–SSZ-13-a during NH_3_–SCR of NO. Initially the sample was held at 150 °C and exposed to a constant stream of gas that comprised; 3000 ppm of NH_3_ and NO in 10% O_2_ at a GHSV of 53,000 h^−1^. The temperature was then increased at 5 °C min^−1^ to 600 °C. The results obtained are shown in Fig. 2. The operando XANES results show that after activation and prior to catalyst activity the spectra contain a small pre-edge shoulder between 8985 and 8990 eV assigned to the Cu^2+^ 1s/4p transition (see Fig. 3a), which is evidence of a move of the Cu^2+^ to a lower symmetry position in the zeolite framework and is indicative of total dehydration and the Cu ions adopting specific crystallographic positions. The temperature at which the catalyst starts to become active (around 200 °C) corresponds to a rapid change in the XANES spectra as a second large pre-edge peak appears with an intense sharp feature at 8982 eV. This feature has been reported to be due to a transition from a 1s to the doubly degenerate 4_pxy_ orbitals in a two-coordinate Cu^+^ system [28–31]. It has been suggested that this feature could explain increased deNOx activity at 200 °C via enabling a fast SCR mechanism [30, 32]. The XANES data presented herein shows that there is a strong temperature dependency regarding the feature at 8982 eV and that heating Cu–SSZ-13-a under standard SCR reaction conditions above 225 °C results in the loss of the Cu^+^ leaving only Cu^2+^ species present. As this temperature corresponds with maximum NO conversion it would suggest that Cu^2+^ is the predominant species present during the standard NH_3_–SCR of NO above temperatures > 200 °C.

Although the feature at 8982 eV rapidly decreases in intensity, it is observed that in the region of maximum catalytic conversion of NO, between 250 and 350 °C there is a small but distinguishable pre-edge peak still present (see Fig. 3a). Although the nature of the Cu(I) has been studied at low NO conversion under standard SCR conditions [33] the cause of the Cu(I) in this temperature regime, which corresponds to maximum NO conversion, has not been adequately ascribed to the mechanism of NH_3_–SCR. To probe this feature further additional isothermal operando experiments were performed at different GHSV, of approximately 100,000 and 225,000 h^−1^ at 250 °C (see Fig. 3b). Curiously in the additional experiments the feature at 8982 eV is not present at 100,000 h^−1^ after 10 min but does start to appear at 20 min exposure. At 225,000 h^−1^ the feature is present after 10 min then increases in concentration by 20 min of exposure however by 70 min exposure this apparent intensity has decreased.

The observed result, of partial Cu(II) reduction to Cu(I), during the initial exposure of the material to reagents, coupled with a change in intensity of the Cu(II) reduction at different GHSV implies that there is a change in the equilibrium of the composition of reactants present in the catalyst, and that at this leads to an environment which can reduce some of the Cu(II) present. The compositional change with in the zeolite material influences the catalytic performance of the material in terms of the conversion of NO. Increasing the GHSV of the gas stream through the catalyst decreases the conversion of NO modestly from 97% at 53,000 h^−1^ to 83% at 225,000 h^−1^ (see ESI for further details), the results are summarised in Table 1. The observed results in the 225,000 h^−1^ experiment, in which the Cu(I) feature intensifies between 10 and 20 min and then decreases dramatically by 70 min exposure, suggests that the material is not under true steady state conditions for a significant amount of time after initial exposure. During this initial period, it can be suggested that NH_3_ is being preferentially adsorbed (due to much stronger adsorbent adsorbate interactions than NO or O_2_) and therefore during this initial period the concentration of adsorbed NH_3_ is much higher than the other components present creating an environment in which Cu(II) can be reduced. It should be noted that the apparent decrease in the pre edge feature intensity between the 100,000 h^−1^ experiment reported here and the 53,000 h^−1^ experiment reported earlier may be due to the different gas compositions used for the experiments at DLS versus the SLS, this was due to the limitations of the minimum flow available to the MFCs at DLS coupled with the gas concentrations meant the minimum quantity of NH_3_ and NO that could be used was 3000 ppm whereas at the SLS the experiments were operated at approximately 1000 ppm of NO and NH_3_ which is closer to conditions present in exhaust streams of lean burn diesel vehicles [22].

**Table 1:** Conversion of NO measured under various conditions reported

Experiment name	GHSV (h^−1^)	Temperature (°C)	Gas conditions	NO conversion (%)
Low temp. low GHSV SCR	53,000	150	3000 ppm of NH_3_, 3000 ppm NO, 100,000 ppm O_2_, make up He	9.09
High temp. low GHSV SCR	53,000	250	3000 ppm of NH_3_, 3000 ppm NO, 100,000 ppm O_2_, make up He	96.92
Low temp. high GHSV SCR	225,000	150	1262 ppm of NH_3_, 1262 ppm of NO, 100,000 ppm O_2,_ make up N_2_	10.60
High temp. high GHSV SCR	225,000	250	1262 ppm of NH_3_, 1262 ppm of NO, 100,000 ppm O_2,_ make up N_2_	82.67

### Further Operando XAS Experiments on Cu–SSZ-13-a

**Fig. 4:**
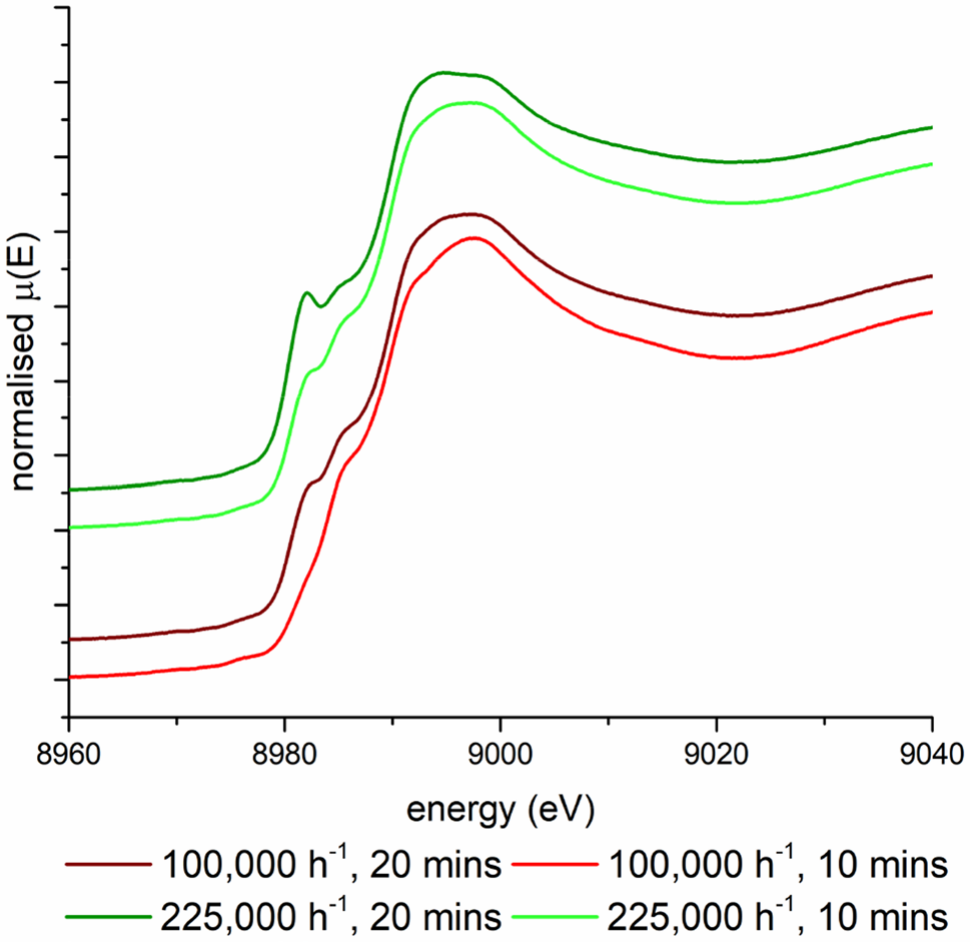
XANES data for Cu–SSZ-13-a under a gas stream containing both NH_3_ and O_2_ conditions showing increased intensity of Cu(I), 1262 ppm of NH_3_, 100,000 ppm O_2,_ make up N_2_ at 250 °C (data recorded at SLS, see ESI for spectroscopic data)

If it is assumed that the stable Cu(I) is independent of the standard SCR reaction, its origin could potentially arise from either the interaction with one of the species present in the gas mixture or through another side reaction for example NO oxidation or NH_3_ oxidation. It has been shown that in Cu–SSZ-13 for NO oxidation to occur it is necessary for the Cu species to be close enough in space to form dimeric species ([Cu–O–Cu]^2+^), [34] at the loadings reported herein (2.92–3.6%) the Cu speciation is below the required Cu loading for NO oxidation to be occurring in a significant amount [35]. Since a significant reduction of the Cu(II) too stable Cu(I) species is observed in the XANES data it was postulated that this may be due to the interaction of the NH_3_ (in the presence of O_2_) and the Cu(II) sites. Therefore, in situ XANES experiments were conducted at appropriate conditions in which NH_3_ and O_2_ were simultaneously present in the gas stream, to probe if the presence of Cu(I) was related to processes involving NH_3_, including adsorption, oxidation or decomposition (see ESI for further details regarding NH_3_ oxidation). It should be noted that the competitive non selective NH_3_ oxidation is usually observed at temperatures significantly higher than 250 °C [36]. Results of the experiments are shown in Fig. 4, in which it is observed that Cu(I) is present at both GHSVs and the intensity is increasing with both increasing GHSV and time on stream.

It has been proposed recently that NH_3_ consumption is a potential route to stable linear Cu(I) species via the following reaction [37]:

1 $${2\text{NH}}_{3} \to {{6\text{H}}^+}+{{\text{N}}_{2}}+{{6\text{e}}^- }$$

2 $${\text{Cu}}{^{2+}}+{{\text{e}}^- } \to {\text{ Cu}}{^+}$$

This would explain the observed features in the XANES data, however, if it were possible for this to be occurring at all Cu species present in the zeolite it should be anticipated that the intensity of the Cu(I) peak present in the XANES spectra would be much greater than is observed (i.e. if all Cu(II) were reduced). A possible explanation for this could be that it has previously been shown that Cu–SSZ-13-a has two different Cu sites occupied in the crystal structure at 250 °C one in the six ring and one in the centre of the eight rings present in chabazite and it may be possible for the NH_3_ activated Cu reduction to be occurring at only one of these sites [18].

### Operando XAS Experiments on Cu–SSZ-13-b

**Fig. 5:**
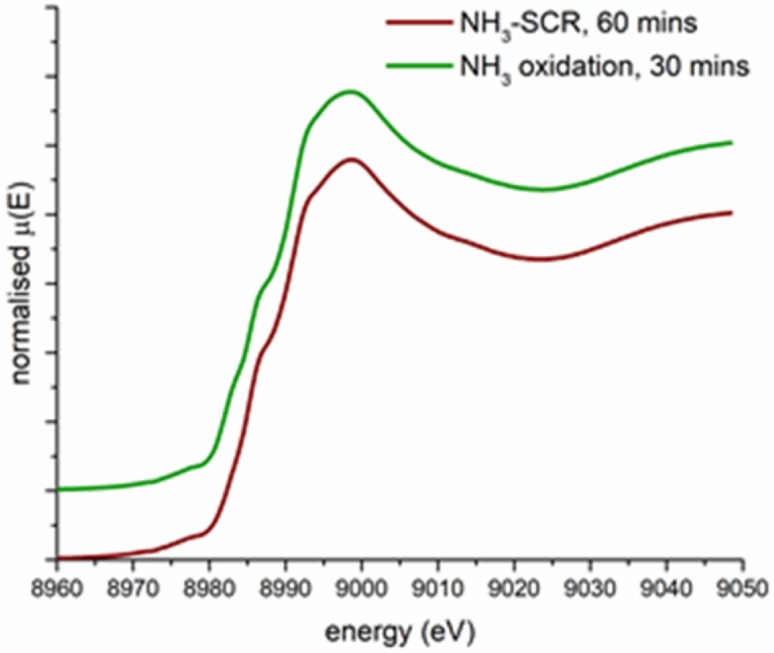
XANES data for Cu–SSZ-13-b under NH_3_–SCR and a gas stream containing both NH_3_ and O_2_ (data recorded at SLS, 1262 ppm of NH_3_, 1262 NO, 100,000 ppm O_2,_ make up N_2_ at 250 °C, GHSV 100,000 h^−1^)

To test this hypothesis Cu–SSZ-13-b was prepared, in which the material has been ion exchanged a further three times and has a lower thermal barrier to Cu migration meaning that at 250 °C all the Cu ions present are in the six ring sites. Cu–SSZ-13-b was tested under conditions for both NH_3_–SCR and NH_3_ + O_2_, the results of these experiments are shown in Fig. 5. Under SCR conditions there is no evidence of Cu(I) species present in the Cu–SSZ-13-b while under the simultaneous exposure of NH_3_ with O_2_ there is only a very small feature centred at 8982 eV present which is far less pronounced than observed under similar conditions for the Cu–SSZ-13-a. These results would suggest that the Cu reduction observed under certain reaction conditions is due to Cu ions present in the eight ring of the chabazite structure, and that by extension, Cu ions present in the six rings cannot undergo NH_3_ mediated reduction. While these results do not rule out the possibility of Cu ions in the eight ring being able to participate in the NH_3_–SCR of NOx the ability for the Cu in the eight ring sites to be reduced through an interaction with NH_3_ may hinder the performance of the 8-ring site relative to the six rings sites in the NH_3_–SCR of NOx.

## Conclusions

Cu–SSZ-13 has been studied in by in operando XANES, the data indicates a role for an interaction between NH_3_ and Cu ions contained within the eight ring of the chabazite structure is responsible for the observed Cu(I) present at temperatures relevant for maximal conversion of NOx (250 °C). During the initial period of exposure to reactants it appears that the concentration of NH_3_ with in the zeolite pores is high enough to create an environment that is capable of reducing the Cu ions contained within the eight ring of the chabazite structure.

Our results highlight the importance of performing both spectroscopic and diffraction studies on catalytic materials as it was only through understanding gained into the migration behaviour of the Cu ions in Cu–SSZ-13-a and Cu–SSZ-13-b [21] that the results reported herein could be rationalised. In the material Cu–SSZ-13-a a small amount of Cu(I) formation is observed under SCR conditions at 250 °C, it has been shown that NH_3_ is responsible for this formation as a sharp feature appears under the conditions in which both NH_3_ and O_2_ are present. Cu–SSZ-13-a is known to have two crystallographically different ion sites occupied at these temperatures, one in the six ring and one in the eight ring, to test if there was any preferential site for NH_3_ consumption the experiments were also conducted on Cu–SSZ-13-b which has been shown to only have Cu occupancy in the 6-ring site at 250 °C. In Cu–SSZ-13-b, there is no evidence of Cu(I) formation during SCR and only a very small feature present under a gas stream containing both NH_3_ and O_2_ (suggesting very low occupancy in the ring of Cu–SSZ-13-b at 250 °C). These results suggest that Cu located in the eight ring sites of chabazite are more reducible than those present in six ring sites, and that at 250 °C Cu located in the eight rings can be readily reduced through an interaction with NH_3_.

The results contained herein could be used to guide the design of the next generation of small pore copper containing zeolites for use in NH_3_–SCR for deNOx applications. Reducing the occupancy of Cu in eight ring under reaction conditions could potentially lead to less Cu(II) reduction, this could either be achieved through changing the Si:Al ratio of the material, the post synthetic ion exchange procedure or through design of zeolite topologies with a higher ratio of 6 ring:8 ring sites.

## Electronic supplementary material

Below is the link to the electronic supplementary material.

Supplementary material 1 (DOCX 775 KB)
