# Supplementary material for: Operando Spectroscopic Studies of Cu–SSZ-13 for NH3–SCR deNOx Investigates the Role of NH3 in Observed Cu(II) Reduction at High NO Conversions
Source: Top Catal. 2018 Jan 19;61(3):175–82. doi: 10.1007/s11244-018-0888-3 (PMC6413821; doi:10.1007/s11244-018-0888-3)
Supplement: Supplementary file 1 — Supplementary material 1 (DOCX 775 KB) [file 11244_2018_888_MOESM1_ESM.docx]

# Supporting information: Operando spectroscopic studies of Cu-SSZ-13 for NH_3_-SCR deNOx investigates the role of NH_3_ in observed Cu (II) reduction at high NO conversions.

A. G. Greenaway^1^, Ines Lezcano-Gonzalez^1^, Miren Agote-Aran^1^, Emma Gibson^1,2^, Yaroslav Odarchenko^1^, Andrew M. Beale^1^

^1^UCL@Harwell, Rutherford Appleton Laboratory, UK

^2^Catalysis Hub, research Complex at Harwell, Rutherford Appleton Laboratory, UK

# Synthesis

The synthesis gel was prepared by hydrolizing tetraethylorthosilicate (20.83 g) and aluminium isopropoxide (1.36 g) in N,N,N-trimethyl-1-adamantamonium hydroxide (79.82 g, 13.2 wt %). The gel mixture is stirred at room temperature overnight until complete evaporation of the alcohol formed during the hydrolysis and the desired water content in the gel was reached. To this gel, Hydrofluoric acid (2.08 g, 48 wt %) is carefully added by dropwise addition the resulting highly viscous mixture is stirred mechanically until a homogenised mixture is achieved. The molar ratio of gel was: SiO_2_ : 0.033 Al_2_O_3_ : 0.50 SDAOH : 0.50 HF : 3 H_2_O where SDAOH is N,N,N-trimethyl-1-adamantamonium hydroxide. The gel is added to a Teflon (44 ml capacity) and sealed tightly into a Parr autoclave, and heated to 150 ^o^C in a static oven. The autoclave is allowed to attain room temperature slowly upon completion of the heating programme, the solid is recovered by vacuum filtration and the washed with copious amounts of water.

The proton form of the zeolite is obtained by calcining the sample in air by heating at 1 °C min^-1^ to 120 °C, held for 2.5 h and then at 4 °C min^-1^ to 580 °C, held for 10 h.

The copper exchanged forms are prepared using a wet ion exchange method; H-SSZ-13 would be added to an aqueous solution of copper sulphate (50 ml of a 0.1 M solution of CuSO_4_ per gram of zeolite) and heated at 80 ^o^C for 2 h with mechanical agitation. The product is then recovered by vacuum filtration washed with copious amounts of water, dried overnight at 80 ^o^C and calcined the sample in air by heating at 1 °C min^-1^ to 120 °C, held for 2.5 h and then at 4 °C min^-1^ to 550 °C, held for 10 h.

# Characterisation

#### PXRD

Pxrd patterns were collected to confirm phase purity and crystallinity on a Rigaku Miniflex diffractometer (Cu K-alpha 1, 1.54056 Å), samples were loaded on to a flat Teflon dish. Diffraction patterns were collected between 5.0 and 50.0 ^o^ in 0.02^o^ steps. PXRD shows that a highly crystalline pure phase of SSZ-13 is present after calcination and subsequent ion exchange calcination steps (See Fig S1). The peak at around 38^o^, present in the collected diffraction patterns is due to the Teflon liner, peak intensities vary from simulated due to preferential orientation and the simulated pattern being from a low Si: Al form containing extra framework K^+^ (instead of Cu^2+^). [S1]





Fig. S1: Simulated PXRD (black), Calcined H-SSZ-13 (red), Cu-SSZ-13-a (1 times Copper exchanged and calcined H-SSZ-13) (blue), and Cu-SSZ-13-b (4 times Copper exchanged and calcined H-SSZ-13) (green) diffraction patterns collected at room temperature in a hydrated state.

#### SEM and EDX

Catalyst morphology was examined by the Scanning Electron Microscopy (SEM). SEM was carried out using JEOL 6610LV outfitted with a tungsten filament. The acceleration voltage of 20 kV was used. Images were acquired using SE detector. SEM EDX analysis was also performed to estimate the Cu weight percent in the catalyst using 80 mm^2^ X-Max silicon drift detector from Oxford instruments.

*Cu-SSZ-13-a: - 1 times Copper exchanged and calcined H-SSZ-13 (See Fig. S2a + b)*


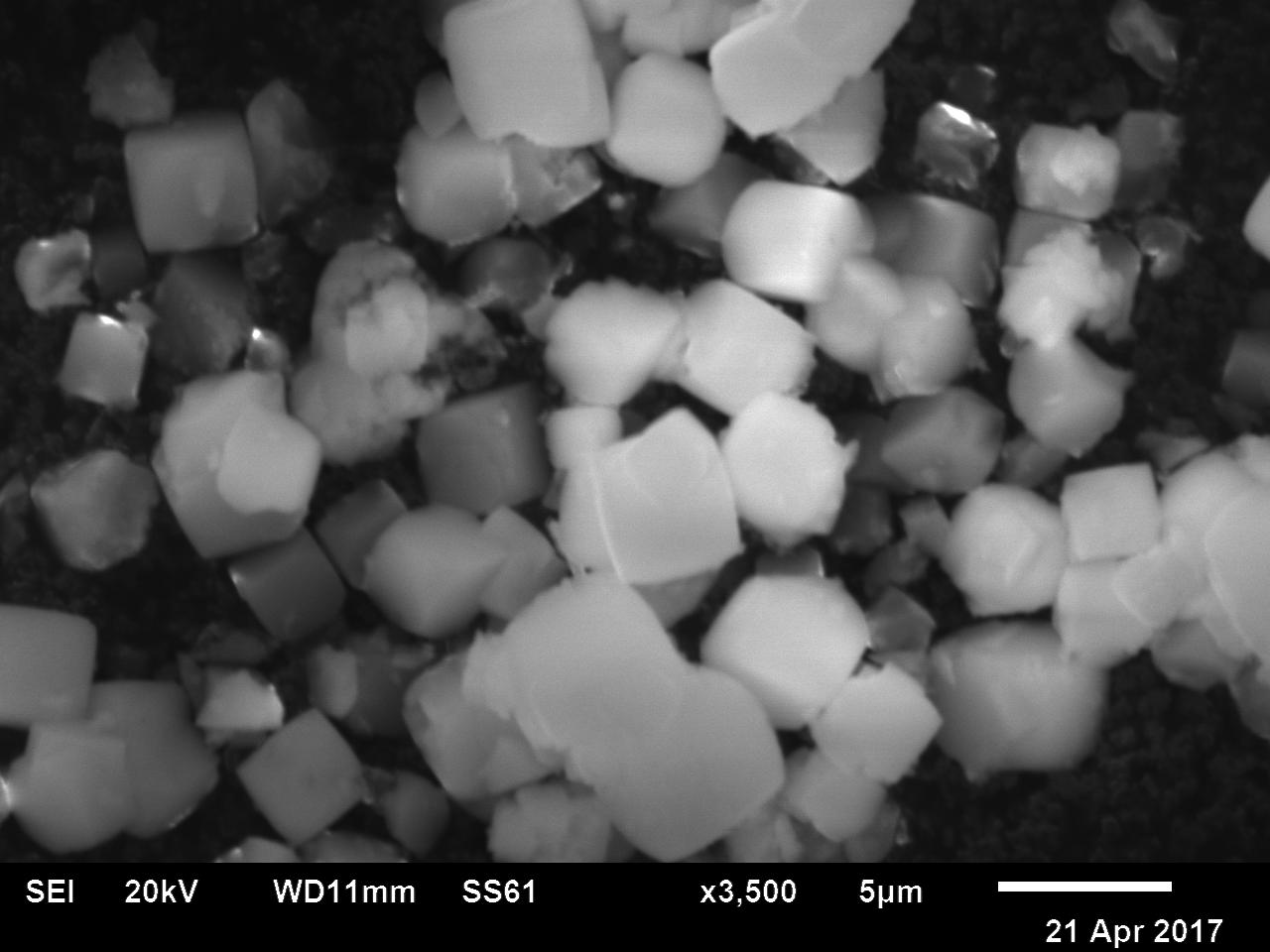


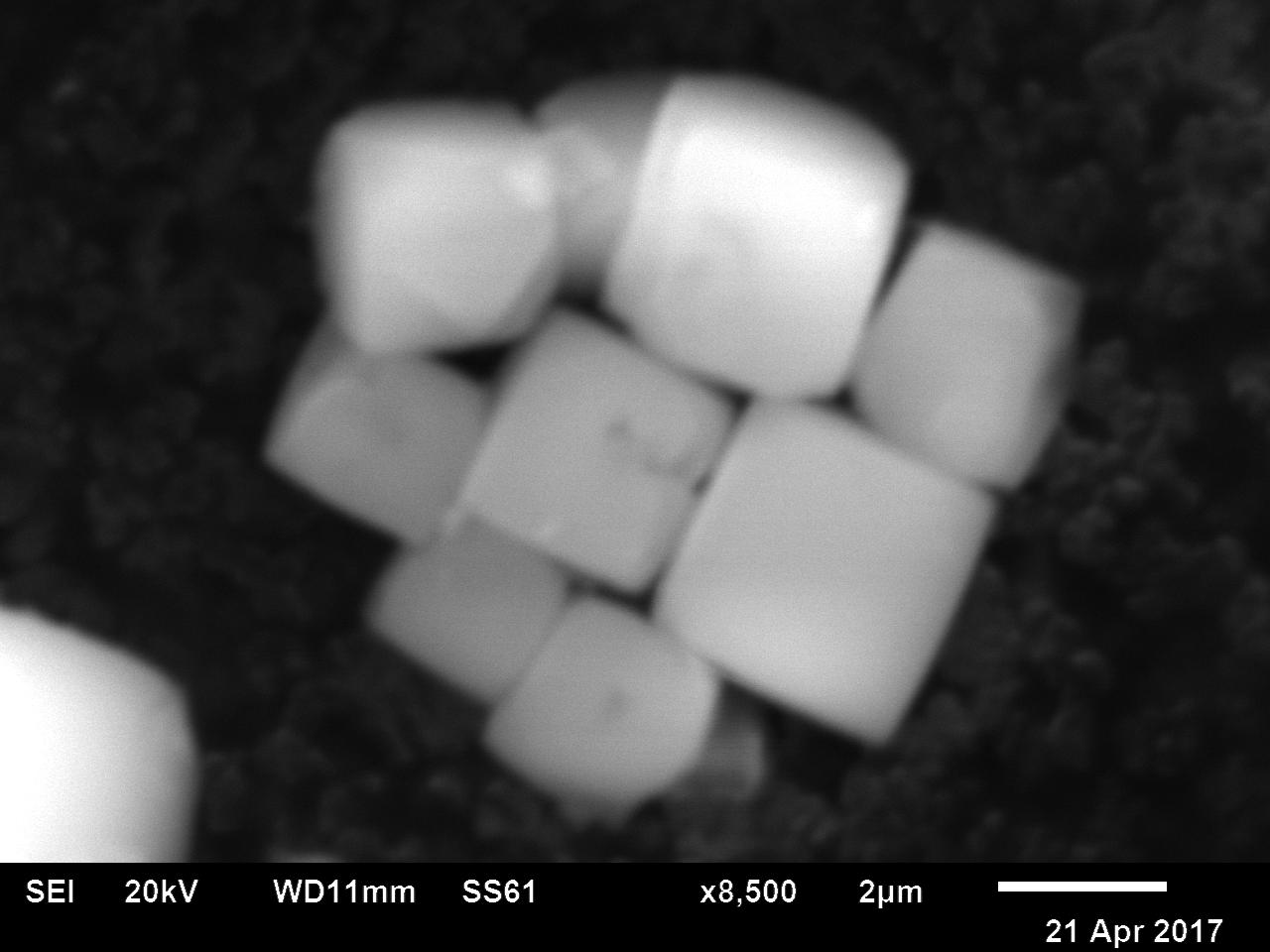


Fig S2: a) wide angle and b) close up SEM back scattered images showing well defined rhombohedral crystals with a size distribution between 2 -5 µm.

*Cu-SSZ-13-b: - 4 times Copper exchanged and calcined H-SSZ-13 (See Fig. S3a + b)*


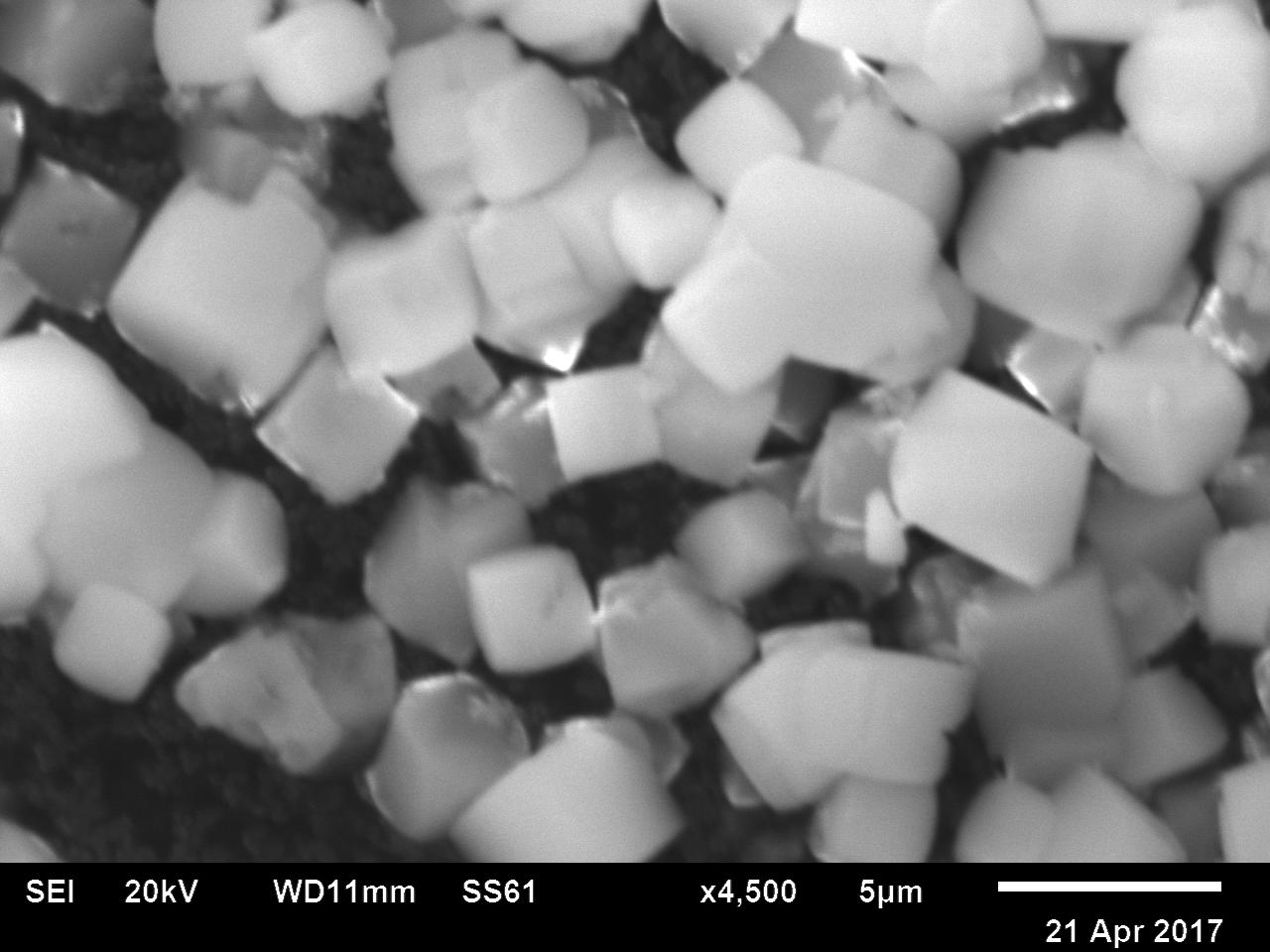

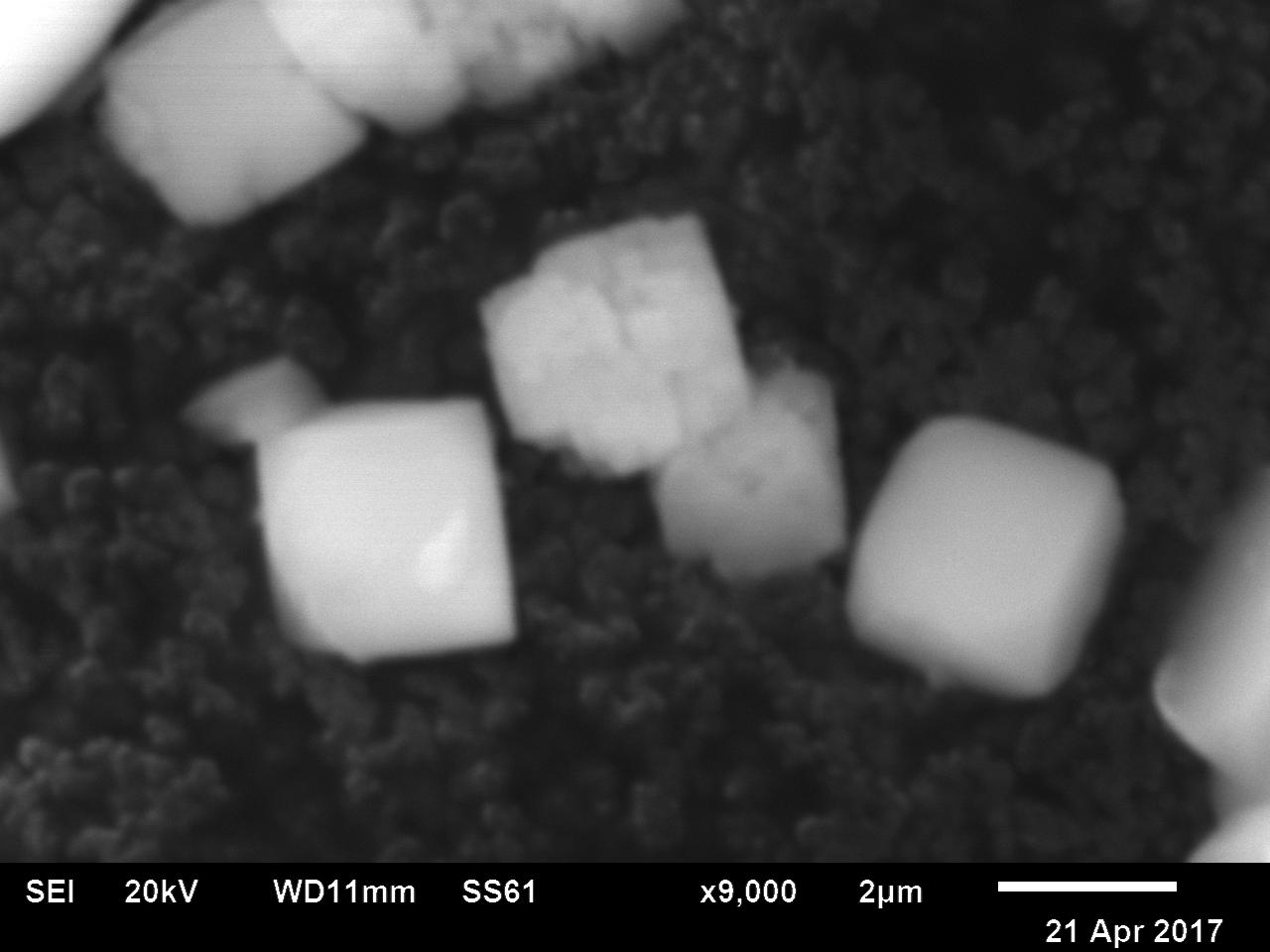


Fig S3: a) wide angle and b) close up SEM back scattered images showing well defined rhombohedral crystals with a size distribution between 2 -5 µm.

EDX analysis of Cu-SSZ-13-a and Cu-SSZ-13-b (See Fig. S4 and S5) and composition of materials table S1 and S2: Cu-SSZ-13-a has a composition of 2.92 wt % Cu with a Si/Al=13 represents 75% Cu ion exchanged in to available H^+^ sites. Cu-SSZ-13-b has a composition of 3.86 wt% Cu with a Si/ Al =12 represents 100% Cu ion exchanged in to available H+ sites.


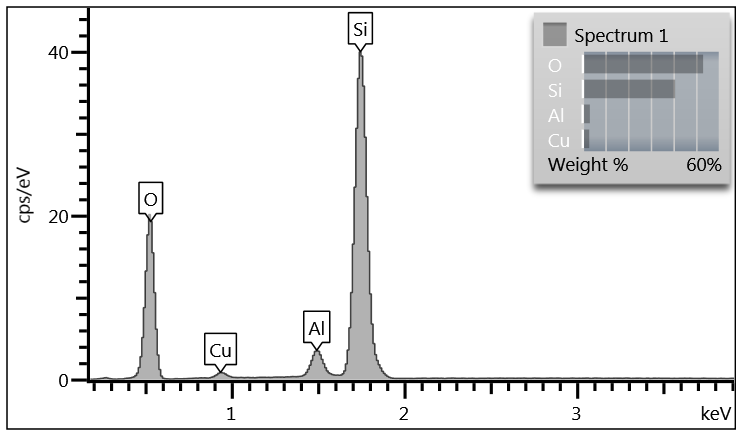


Fig S3: EDX of Cu-SSZ-13-a

Table S1: composition analysis of Cu-SSz-13-a

| Element | Wt% | Wt% Sigma |
| --- | --- | --- |
| O | 53.12 | 0.15 |
| Al | 3.19 | 0.04 |
| Si | 40.77 | 0.13 |
| Cu | 2.92 | 0.10 |
| Total: | 100.00 |  |


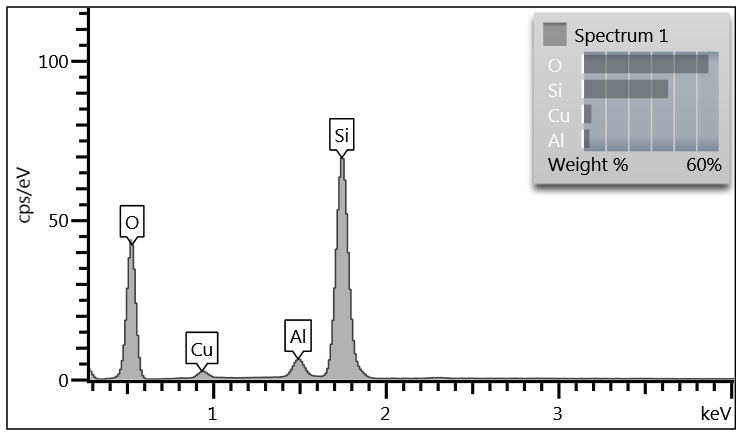


Fig S4: EDX of Cu-SSZ-13-b

Table S2: composition analysis of Cu-SSz-13-b

| Element | Wt% | Wt% Sigma |
| --- | --- | --- |
| O | 55.45 | 0.10 |
| Al | 3.03 | 0.03 |
| Si | 37.67 | 0.09 |
| Cu | 3.86 | 0.07 |
| Total: | 100.00 |  |

#### UV VIS

Diffuse reflectance UV visible (DRUV) spectra were recorded of samples dehydrated in situ on using a Carry 4000 spectrometer (Agilent). All spectra were recorded in the range 200-800 nm and are presented in Kubelka-Munk units , F(R)=(1-R)2/2R, where R is the absolute reflectance of the sampled layer. Samples were loaded into a modified DRIFTS cell and loaded in to the spectrometer. Samples were heated at 400 ^o^C under flowing oxygen (10% in N_2_) to fully dehydrate the materials (see Fig. S4). Samples were then allowed to attain room temperature, and spectra recorded. Back ground was subtracted using a dehydrated form of H-SSZ-13. Upon dehydration samples change from a light blue to intense dark blue colour. This is shown in the UV-vis spectra as a strong blue shifted adsorption in the visible region of the spectrum, consistent with a Cu(II) environment adopting a lower coordination / symmetry (less Laporte forbidden). This feature is observed as a broad and asymmetric absorption in the UV-Vis spectrum around 16660 cm^-1^ and is due to a ^2^E_g_ and ^2^T_2g_ transition; the asymmetry arising due to the Jahn–Teller distortion be resolved into three sub-components at approximately 20000, 16660 and 13330 cm^-1^ which corresponding to transitions originating from d(xz), d(yz) - d(x^2^ - y^2^ ), d(xy) - d(x^2^ - y^2^ ) and d(z ^2^ ) - d(x^2^ - y^2^ ). These features all indicate that there are isolated Cu^2+^ ions present in the dehydrated sample. Importantly the spectra lack other features associated with [Cu–O–Cu]^2+^ dimers and [Cu2(μ-η^2^:η^2^-O_2_]^2+^ dimer which have been previously observed in other materials. [S2]





Fig. S4:- DRUV spectra collected for Cu-SSz-13-a (light blue) and Cu-SSZ-13-b dark blue. Samples were collected at room temperature after being activated at 400 ^o^C under flowing O_2_ in N_2_ (10%)

#### Catalytic testing


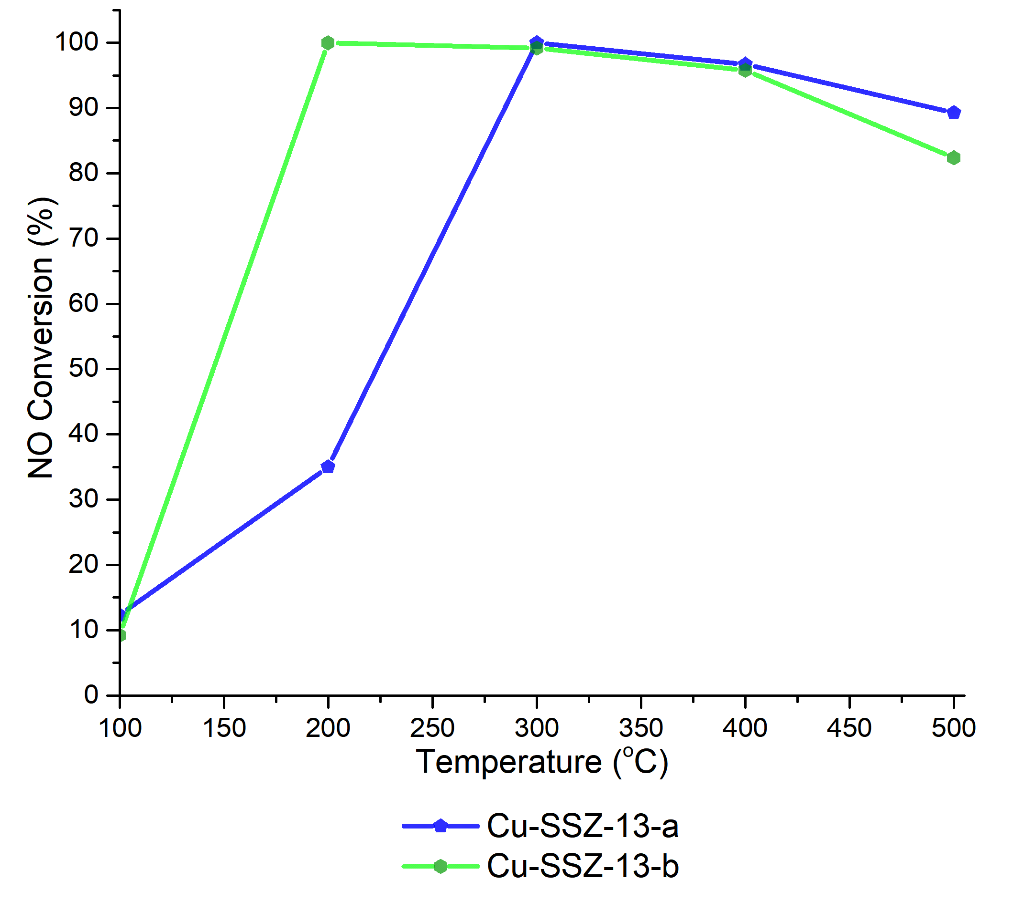


Fig. S5. NO conversion for Cu-SSZ-13-a and Cu-SSZ-13-b ay a GHSV of 100 000 h-^1^, feed composition of 1000 ppm NO, 1000 ppm NH_3_ and 5% O_2_, make up N_2_. NO conversion was calculated as a function of intensity of NO mass spec signal and the intensity of equilibrated NO mass spec signal at low temperature (((I_re_f-I_temp_)/I_ref_) x 100 %). NH_3_ SCR de-NOx of NO was conducted at several temperatures between 100 and 500 ^o^C, the NO conversions calculated are reported below.

# Gas Hourly Space Velocity Calculations

#### DLS operando XANES

Volume of catalytic bed = 56.55 mm^3^

Total flow = 50 ml per minute

GHSV = 53051.7 h^-1^

Gas composition:-

| Gas | Composition (% active gas in He) | ppm active gas | Desired ppm | percent total of stream | total stream (ml min^-1^) |
| --- | --- | --- | --- | --- | --- |
| NO in He | 1 | 10000 | 3000 | 30 | 15 |
| NH_3_ in He | 5 | 50000 | 3000 | 6 | 3 |
| O_2_ in He | 17.5 | 175000 | 100000 | 57.1 | 28.6 |
| He | 100 |  |  | 6.9 | 3.4 |

#### SLS operando XANES

Medium GHSV (approx. 100 000 h^-1^)

Volume of catalytic bed = 13.3 mm^3^

Total flow = 19.8 ml per minute

GHSV = 89323.3 h^-1^

Gas composition: -

| Gas | Composition (% active gas in N_2_) | ppm active gas | Desired ppm | percent total of stream (%) | total stream (ml min^-1^) |
| --- | --- | --- | --- | --- | --- |
| NO in N_2_ | 1 | 10000 | 1262 | 12.63 | 2.5 |
| NH_3_ in N_2_ | 1 | 10000 | 1262 | 12.63 | 2.5 |
| O_2_ | 100 | 1000000 | 101010 | 10.1 | 2 |
| N_2_ | 100 |  |  | 64.7 | 12.8 |

High GHSV (approx. 225 000 h^-1^)

Volume of catalytic bed = 13.3 mm^3^

Total flow = 49.5 ml per minute

GHSV = 223308.3 h^-1^

Gas composition: -

| Gas | Composition (% active gas in N_2_) | ppm active gas | Desired ppm | percent total of stream | total stream (ml min^-1^) |
| --- | --- | --- | --- | --- | --- |
| NO in N_2_ | 1 | 10000 | 1262 | 12.63 | 6.25 |
| NH_3_ in N_2_ | 1 | 10000 | 1262 | 12.63 | 6.25 |
| O_2_ | 100 | 1000000 | 101010 | 10.1 | 5 |
| N_2_ | 100 |  |  | 64.7 | 32 |

# NH_3_ oxidation and possible mechanism

It has been shown that as temperature increases NH_3_ oxidation via the direct oxidation with oxygen becomes competitive with standard SCR, the proposed reactions involved in NH_3_ oxidation by oxygen include: - [S3]

2 NH_3_ + 3/2 O_2_ → N_2_ +3 H_2_O (1)

2 NH_3_ + 2 O_2_ → N_2_O +3 H_2_O (2)

2 NH_3_ + 5/2 O_2_ → 2NO +3 H_2_O (3)

Under high temperature SCR, the reactions proposed account for unequal (non-stoichiometric) conversion of NO and NH_3_. In general reaction (1) is more favoured at lower temperatures whereas reaction (3) becomes more favourable at higher temperatures. Reaction (1) is a potential route to prevent NH_3_ slip without the need to add additional components in to the exhaust stream.

# SLS Operando experiments out gas analysis

Fig. S6. Mass spec analysis of Cu-SSZ-13-a, during NH_3_ SCR of NO under high GHSV (225 000 h^-1^) conditions at SLS.


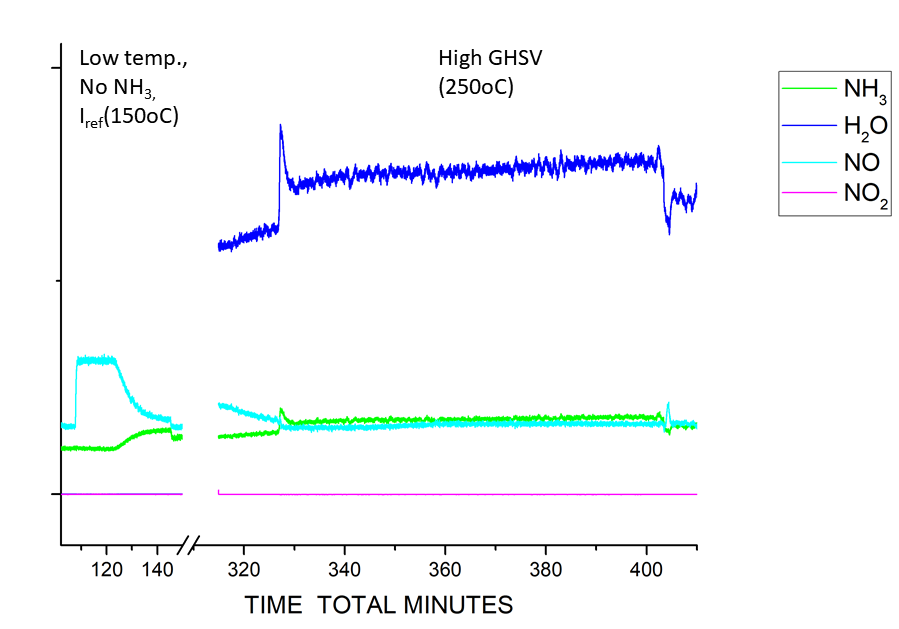


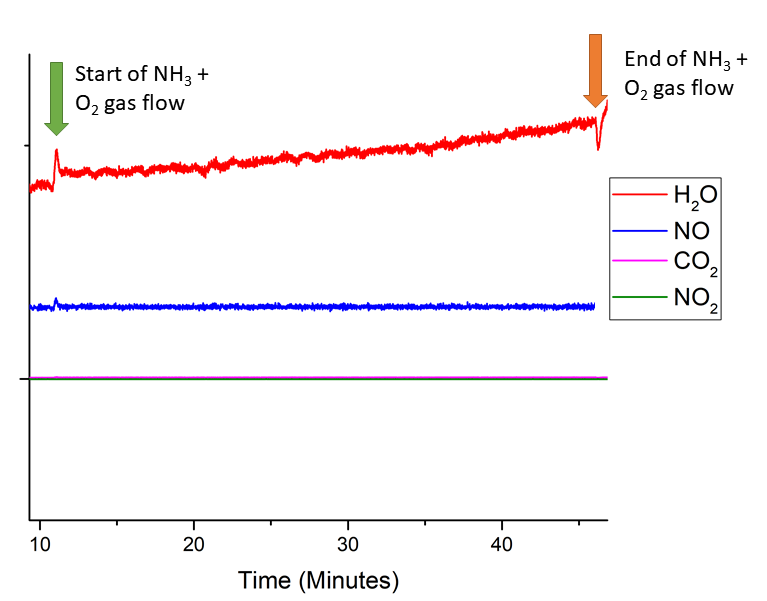


Fig. S7. Mass spec analysis of Cu-SSZ-13-a, during NH_3_ +O_2_ flow conditions under high GHSV (225 000 h^-1^) conditions at SLS

# References

[S1] Calligaris, M., Nardin, G., Randaccio L., (1983) *Zeolites*, 3, 205-208

[S2] Giordanino F., Vennestrøm P.N.R., Lundegaard L.F, Stappen F.N., Mossin S., Beato P., Bordiga S., Lamberti C., (2013) *Dalton Trans*., 42, 12741

[S3] Busca G., Lietti L., Ramis G., Berti F., (1998) *Applied Catalysis B: Environmental* 18, 36
